# Consistency and timeliness of intrapartum care interventions as predictors of intrapartum stillbirth in public health facilities of Addis Ababa, Ethiopia: a case-control study

**DOI:** 10.11604/pamj.2021.40.36.25838

**Published:** 2021-09-14

**Authors:** Alemayehu Gebremariam Agena, Lebitsi Maud Modiba

**Affiliations:** 1 Global Health Delivery, University of Global Health Equity (UGHE), Kigali, Rwanda,; 2 Department of Health Studies, 160 College of Human Sciences, University of South Africa, Pretoria, South Africa

**Keywords:** Intrapartum stillbirth, labour, Addis Ababa, live birth

## Abstract

**Introduction:**

approximately one-third of the global stillbirth burden occurs during intrapartum period. Intrapartum stillbirths occurring in the health facilities imply that a foetus was alive on admission to labour and had greater chances of survival with optimum obstetric care. Active monitoring and follow-up by skilled birth attendants becomes critical to determine the progress of labour and to decide any emergency obstetrical care actions. Timely monitoring of labour progress indicators including fetal heart rate (FHR), uterine contraction maternal vital signs, vaginal examination (VE) are vital in reducing intrapartum stillbirth.

**Methods:**

a case-control study was conducted using primary data from chart review of medical records of women who experienced intrapartum stillbirth in 20 public health centres and 3 public hospitals of Addis Ababa between July 1^st^, 2010 to June 30^th^, 2015. Data were collected from charts of all cases of intrapartum stillbirths meeting the inclusion criteria and randomly selected charts of controls from each public health facility in 2: 1 control to case ratio.

**Results:**

over 90% of both cases and controls received FHR monitoring care albeit the timing was substandard. More women in the live birth group than intrapartum stillbirth group received timely care related to uterine contraction (OR 2.42, 95% CI 1.77 - 3.30) and blood pressure monitoring (aOR 1.41, 95% CI 1.09 - 1.81). 1.2% and 0.3% of women in the intrapartum stillbirth and livebirth groups developed eclampsia respectively.

**Conclusion:**

substandard timing and application of labour monitoring interventions including FHR, uterine contraction can predict intrapartum stillbirth in public health facilities.

## Introduction

Intrapartum stillbirth is defined as the delivery of any foetus after 28 weeks of gestation, or with a birth weight more than 1000g, who had detectable foetal heart sounds upon admission, but died during the intrapartum period and therefore had an Apgar score of 0 at 1 and 5 min, without signs of maceration [1]. Evidence shows that approximately 1.3 million intrapartum stillbirths occur in the world annually. This magnitude accounts for half of all stillbirths occurring globally. However, the rate of intrapartum stillbirth is higher in low-resource settings such as sub-Saharan Africa [2]. A study from India revealed that intrapartum period associated cause of stillbirth was as high as 48.3% [3].

Where women receive quality intrapartum care, as in many high-income countries, the proportion of intra-partum stillbirths is less than 10% of all stillbirths, indicating that a substantial proportion of intrapartum stillbirths are preventable with quality intrapartum care [4]. Intrapartum stillbirth occurring in the health facilities implies that a foetus was alive on admission to labour and could have survived if timely care was offered. Given the advancement in medicine, obstetrics and medical technology, intrapartum stillbirth in a health facility can be reduced significantly. Proper investment both on the demand and supply side of obstetric care services are critical to redress this most neglected tragedy in global health [5]. To this effect, high quality intrapartum interventions focusing on effective management of maternal and foetal risk factors that cause intrapartum stillbirth are critical to reduce the burden [6].

Whilst specific labor management protocols can vary from country to country, many developing countries including Ethiopia employ partograph as a tool to monitor the fetal and maternal wellbeing and progress of labor in health facilitates. Partograph offers recommended time intervals for each indicator and results should be recorded in the respective spaces on the graph to help with decision-making on the course of obstetric actions. For instance, fetal heartbeat and maternal contractions should be measured every 30 minutes and cervical dilatation should be assessed every 4 hours. Labor management deviating from these recommended types and timing of labor monitoring indicators can be referred as substandard [7]. This study collected data from the public health facility in Addis Ababa on key labour monitoring interventions including FHR, maternal vital signs, uterine contraction, vaginal examination, management of labour complications for the index pregnancy to see if any of these had statistically significant associations with intrapartum stillbirth.

## Methods

### Definitions

**Partograph:** is a chart on which the salient features of labour are entered in a graphic form and therefore provides the opportunity for early identification of deviations from normal. The charts are usually designed to allow for recordings at 15 minutes intervals and include: foetal heart rate; maternal temperature; pulse; blood pressure; details of vaginal examinations; strength of contractions; frequency of contractions in terms of the number in 10 min; fluid balance; urine analysis and drugs administered [8].

**Optimum time to assess labour progress monitoring indicators:** data form the maternity charts were categorized as optimum timing if labour monitoring were conducted every half hourly for foetal heartrate, uterine contraction and maternal pulse measurements. Similarly, records were considered optimum if vaginal examination, maternal blood pressure and temperature were assessed every four hours [9-12].

**Sub-standard timing of labour progress monitoring:** any labour monitoring assessment that does not meet the definition of optimum timing as indicated in the above definition is considered as substandard timing in this study.

**Study setting and design:** this was a case-control study that was conducted using primary data from chart review of medical records of women who experienced intrapartum stillbirth in 20 public health centres and 3 public hospitals of Addis Ababa during the period July 1^st^, 2010 to June 30^th^, 2015. In 2010, 26 public health centres offered basic emergency obstetric and neonatal care (BEmONC) in Addis Ababa [13] out of which 20 were selected for this study based on availability of data. Similarly, chart reviews were conducted in three out of the five public hospitals under the Addis Ababa city administration, where comprehensive emergency obstetric and neonatal care (CEmONC) had been practiced since 2010. Six health centers and three public hospitals were not included because obstetric care services began in these facilities after the reference baseline. BEmONC is a minimum packages of seven essential obstetric care services including parenteral administration of antibiotics, uterotonic drugs, general anticonvulsants; and performing manual removal of placenta, retained products, assisted delivery and basic neonatal resuscitation [14]. CEmONC consists of all obstetric interventions under BEmONC package, in addition to two specialized services including caesarean section and blood transfusion which are usually provided in the tertiary health facilities [15].

**Study population and sampling:** all cases of intrapartum stillbirth that were occurred in the 20 public health centres and 3 public hospitals in Addis Ababa and recorded in the maternity registers of respective facilities were considered for this study. Given intrapartum stillbirth is a relatively rare phenomenon, this study included all cases of intrapartum stillbirths meeting the inclusion criteria. Intrapartum stillbirth in this study refers to babies who were alive during admission for labour in public health facilities but pronounced dead on delivery as was registered in the maternity medical records. Controls were selected randomly from the same maternity registers which served as a sampling frame in each public health facility. On every page of the maternity registers where cases of intrapartum stillbirth were taken, record numbers of women with livebirth were listed and rolled on pieces of paper of which an individual other than the data collector randomly selected until the required sample was obtained to achieve the two to one (2: 1) control to case ratio.

**Sample size:** accordingly, of the documented 112 intrapartum stillbirth cases in the 20 public health centres in Addis Ababa, 91 (81%) met the selection criteria and were included in this study. Similarly, there were a total of 944 cases of intrapartum stillbirth in the three public hospitals of which 637 (67%) qualified the inclusion criteria including completeness of medical record, foetal heart rate on admission for labour and the birth assisted by qualified health workers in a public health facility. A total of 427 chart of controls were reviewed in the 20 public health centres of which only 273 (64%) were included. Moreover, 1738 controls were also randomly identified in the three public hospitals of which 1278 (74%) qualified the inclusion criteria. The charts of control groups were selected from the registers in a random manner using lottery method. On every page where cases of intrapartum stillbirth were detected, record numbers of women with livebirth were listed from the pages of registers where cases are picked and rolled on pieces of paper of which an individual other than the data collector randomly selected the required number of controls. In general, 728 cases of intrapartum stillbirth and 1551 controls were reviewed for this study.

**Data collection and analysis:** quantitative data on key variables related to labour monitoring interventions were collected from the obstetric records of women who had given birth in the public health facilities in Addis Ababa from July 1^st^, 2010 to June 30^th^, 2015. Data entry and analysis were conducted using SPSS version 24 from August 1^st^ to Sept 30^th^, 2016. Bivariate analysis was conducted for key independent variables followed by multivariate logistic regression model for variables with p-value of 0.2 and less.

**Ethical considerations:** data were collected from medical records where information from individual charts were de-identified thereby minimising the concerns of confidentiality and requirements for individual consents. The data collector was trained and strictly monitored on the principles of confidentiality of clients' information on the records during the process of data collection. The chart review was conducted within the respective facilities through consented authorisation of relevant facility leadership. Individual data sources remained anonymous during analysis and report writing. Furthermore, ethical approval was obtained from the Higher Degrees of the University of South Africa (HSHDC/421/2015), and study permit was secured from health ethics committee of Addis Ababa Regional Health Bureau (AARHB) prior to data collection.

## Results

**Socio-demographic characteristics:** data were collected on five key socio-demographic variables including age, marital status, gravida, para and number of children alive for the women whose charts were reviewed in this study. Table 1 presents that approximately 57% of women who experienced intrapartum stillbirth and 60% who had livebirths reported to be in the age category 25-34 years. The second highest proportion of women in the study population for both intrapartum stillbirth (35.8%) and livebirth (33.6%) were found in the age group 15-24 years. Results from this study showed that proportionally more women in the stillbirth category (49.3%) than in the livebirth (37.1%) conceived for the first time. This study did not reveal any statistically significant association between intrapartum stillbirth and birth order (Table 1).

**Table 1:** key socio-demographic characteristics affecting intrapartum stillbirth

Characteristic	Stillbirth N (%)	Livebirth N (%)	p-value
**Age (years)**			
15-24	261 (35.8)	522 (33.6)	0.333
25-34	416 (57.2)	931 (60.3)	
35-49	51 (7.0)	98 (6.1)	
**Marital status**			
Married	314 (42.7)	982 (64.4)	0.386
Divorced	3 (0.4)	5 (0.3)	
Widowed	0 (0.0)	3 (0.2)	
Separated	0 (0.0)	2 (0.1)	
Never married	11 (1.5)	43 (2.8)	
Missing	400 (54.9)	516 (33.2)	
**Gravida**			
One	360 (49.3)	575 (37.1)	0.000
Two	203 (28.0)	539 (34.8)	
Three	84 (11.5)	256 (16.5)	
Four	55 (7.6)	133 (8.6)	
Five and above	26 (3.7)	48 (3.0)	
**Para**			
Zero	442 (60.3)	744 (48.1)	0.000
One	185 (25.4)	542 (35.0)	
Two	57 (7.9)	177 (11.4)	
Three	31 (4.3)	61 (3.9)	
Four	10 (1.5)	19 (1.2)	
Five and above	4 (0.5)	8 (0.5)	
**Children**			
Zero	451 (68.8)	790 (55.2)	0.000
One	134 (20.4)	435 (30.4)	
Two	43 (6.6)	139 (9.7)	
Three	21 (3.2)	49 (3.4)	
Four and above	7 (1.1)	17 (1.2)	
Missing	72 (9.8)	121 (7.8)	

**Types and timing of intrapartum care:** findings from this study revealed that approximately 85% women in the stillbirth and 98% in the livebirth groups had foetal heartbeat between 110- 160/min on admission to labour units in the health facilities. The results further showed that over 99% of women in both intrapartum stillbirth and livebirth groups were assessed for FHR during childbirth without any statistically significant differences between the two groups. However, 99% of women both in the intrapartum stillbirth and livebirth categories did not receive timely FHRM; every 30 minutes as recommended for labour management using a partograph.

Proportionally, more women in the livebirth category (94.6%) than in the stillbirth category (87.8%) were offered any care related to monitoring uterine contraction during labour in review. Women who did not receive intrapartum care related to uterine monitoring were over twice more likely to experience intrapartum stillbirth compared to those who received the service (OR 2.42, 95% CI 1.77-3.30). The difference between the two groups was statistically significant (p<0.01). Similarly, larger proportion of women in the intrapartum stillbirth category (87.5%) than in the livebirth group (79%) did not receive monitoring of uterine contraction within the recommended time intervals. Conversely, only 12.5% women in the intrapartum stillbirth category against 20.9% women in the livebirth category received timely monitoring of uterine contraction, the difference being statistically significant (p<0.01). Furthermore, higher proportion of women in the stillbirth group (12.2%) than in the livebirth group (6.4%) had missing records regarding uterine contraction monitoring during the intrapartum period.

Furthermore, proportionally more women in the livebirth category (92.7%) against women who experienced intrapartum stillbirth (89.7%) received care related to monitoring blood pressure during labour, the difference being statistically significant (p=0.02). Nevertheless, the timing of blood pressure monitoring was not consistent with standard for 64.4% of women in the intrapartum stillbirth category compared to 62.5% in the livebirth group. This study collected clinical data from intrapartum records of both cases and controls to assess if VE were provided routinely as per the recommended frequency for women given birth in the public health facilities in Addis Ababa. Accordingly, more than 99.5% of women in both groups received VE in the respective health facilities during the index childbirth. However, proportionally more women in the livebirth group (49.5%) than in the intrapartum stillbirth group (44.2%) received VE as per the recommended intervals during the index labour (p=0.02). More importantly, a higher proportion of women in the stillbirth group (39.7%) had missing data on the interval of VE compared to women in the livebirth category (2.5%).

The five-year data from the public health facilities in Addis Ababa indicate that 27.6% of women in the intrapartum stillbirth category received episiotomy compared to 32.4% in the livebirth group (p=0.02) (Table 2). Results from the logistic regression analysis revealed important associations between key obstetric interventions during labour and birth outcome. For instance, women who received uterine contraction monitoring inconsistently were at an increased risk of having intrapartum stillbirth (AOR 1.55, 95% CI 1.09-2.18). Women who did not receive BP monitoring were 1.4 times more likely to experience intrapartum stillbirth compared to those who received the service (AOR 1.44, 95% CI 1.07-1.96). Furthermore, results from this study showed statistically significant differentials in timing of VE where women in the intrapartum stillbirth group received substandard monitoring (AOR 1.41 95% CI 1.09 - 1.81) compared to their counterparts in the livebirth group. Similarly, women who did not receive episiotomy during the index delivery were 1.5 times more likely to experience intrapartum stillbirth (AOR 1.51, 95% CI, 1.15-1.97) thereby making the service one of the determinants of intrapartum stillbirth (Table 3).

**Table 2:** types and timing of intrapartum care during the index pregnancy in the public health facilities

Interventions	Categories	Stillbirth N (%)	Livebirth N (%)	P-value
FHRM care given	Yes	727 (99.9)	1549 (99.9)	0.434
	No	1 (0.1)	2 (0.1)	
Timing of FHRM - 15 min care consistent	Yes	3 (0.4)	12 (0.8)	0.870
	No	725 (99.6)	1539 (99.2)	
Uterine contraction monitoring	Yes	638 (87.8)	1464 (94.6)	0.000
	No	90 (12.2)	87 (5.4)	
Timing of uterine contraction monitoring	Yes	80 (12.5)	304 (20.9)	0.000
	No	559 (87.5)	1148 (79.1)	
	Missing	89 (12.2)	99 (6.4)	
Maternal blood pressure (BP) care given	Yes	652 (89.7)	157 (92.6)	0.018
	No	76 (10.3)	114 (7.4)	
Timing of maternal blood pressure (BP)	Yes	232 (35.6)	532 (37.5)	0.417
	No	419 (64.4)	887 (62.5)	
	Missing	77 (10.6)	132 (8.5)	
Maternal temperature care given	Yes	50 (6.8)	82 (5.3)	0.156
	No	678 (93.2)	1469 (94.7)	
Timing of maternal temperature	Yes	20 (37.7)	37 (42.5)	0.576
	No	33 (62.3)	50 (57.5)	
	Missing	675 (92.7)	1464 (94.4)	
Maternal pulse care given	Yes	447 (61.2)	992 (64.0)	0.191
	No	281 (38.8)	559 (36.0)	
Timing of maternal pulse care consistent	Yes	52 (11.4)	132 (13.2)	0.329
	No	404 (88.6)	865 (86.8)	
	Missing	272 (37.4)	554 (35.7)	
Vaginal examination (VE) care given	Yes	726 (99.7)	1539 (99.5)	0.405
	No	2 (0.3)	12 (0.5)	
Timing of vaginal examination	Yes	321 (44.2)	749 (49.5)	0.019
	No	407 (55.8)	764 (50.5)	
	Missing	289 (39.7)	38 (2.5)	
Oxytocin care provided	Yes	100 (14.1)	235 (15.0)	0.601
	No	628 (85.9)	1316 (85.0)	
Episiotomy care conducted	Yes	201 (27.6)	497 (32.4)	0.020
	No	527 (72.4)	1054 (67.6)	
Vacuum/forceps delivery care given	Yes	53 (7.3)	123 (8.0)	0.543
	No	675 (92.7)	1428 (92.0)	

**Table 3:** key results from logistic regression on intrapartum care during the index pregnancy

Independent variable	Birth outcome		Crude OR (95% CI)	Adjusted OR (95% CI)
	**Stillbirth N (%)**	**Live birth N (%)**		
**Gravida**				
One	363 (49.3)	573 (37.1)	1.1 (0.67-1.8)	
Two	206 (28.0)	537 (34.8)	0.67 (0.41-1.1)	
Three	85 (11.5)	254 (16.5)	0.58 (0.34-0.99)	
Four and above	83 (11.3)	179 (11.6)	1	
**Para**				
Zero	444 (60.3)	741 (48.1)	1.19 (0.35-4.00)	
One	187 (25.4)	539 (35.0)	0.69 (0.21-2.33)	
Two	58 (7.9)	176 (11.4)	0.66 (0.19-2.27)	
Three	32 (4.3)	60 (3.9)	1.06 (0.29-3.82)	
Four and above	15 (2.0)	26 (1.7)	1	
**Children alive**				
Zero	451 (68.8)	790 (55.2)	1.78 (1.47-2.17)	1.48 (1.12-1.95)
One or more	205 (31.2)	640 (44.8)	1	1
**Uterine contraction monitored**				
Yes	646 (87.8)	1458 (94.6)	1	1
No	90 (12.2)	84 (5.4)	2.42 (1.77-3.30)	0.37 (0.03-4.13)
**Timing of uterine contraction observation consistent**				
Yes	80 (12.5	304 (20.9)	1	1
No	559 (87.5)	1148 (79.1)	1.85 (1.42-2.42)	1.55 (1.09-2.18)
**Maternal blood pressure (BP) monitored**				
Yes	659 (89.7)	1427 (92.6)	1	1
No	76 (10.3)	114 (7.4)	1.44 (1.07-1.96)	1.02 (0.62-1.65)
**Timing of vaginal examination (VE) care consistent**				
Yes	321 (44.2)	749 (49.5)	1	1
No	405 (55.8)	764 (50.5)	1.24 (1.04-1.48)	1.41 (1.09-1.81)
**Episiotomy care conducted**				
Yes	201 (27.6)	497 (32.4)	1	1
No	528 (72.4)	1037 (67.6)	1.26 (1.04-1.53)	1.51 (1.15-1.97)

## Discussion

In this study labour monitoring interventions including FHR, uterine contraction, maternal blood pressure, vaginal examination and episiotomy care were key determinants associated with intrapartum stillbirth. In this study, over 99% of women in both stillbirth and livebirth groups were assessed for FHR during labour however the intervention was not consistent with recommended intervals. FHR monitoring practice was more common in this study compared to similar studies from Zanzibar and Nepal where the rate among women who experienced intrapartum stillbirth were 50% and 25% respectively [16,17]. However, this finding was not consistent with a study from Tanzania where proportionally more women (83%) in the intrapartum stillbirth category did not receive timely fetal heart rate monitoring (FHRM) compared to women in the livebirth category (67%) [16].

Women who had inconsistent monitoring of uterine contraction were 1.5 times more likely to experience intrapartum stillbirth. This finding is consistent with a study from Zanzibar where proportionally lower women in the intrapartum stillbirth group than controls had their uterine contractions monitored within the recommended time interval [16]. The finding has strong policy significance to improve the quality of obstetric care and to reinforce the implementation of standard protocols by service providers in health facilities [18].

Consistent monitoring of maternal blood pressure (BP) during intrapartum period as per the recommended clinical standard would save lives [8]. Given pre-eclampsia can affect up to 2.9% of pregnancies, close and timely monitoring of maternal blood pressure during labour is considered a good obstetrical practice that could save lives [19]. Accordingly, women who didn´t receive BP monitoring during labour had 1.4 times higher chance of having intrapartum stillbirth. Similarly, increased temperature during labour could cause obstetric complications or adverse outcomes. A study in the USA indicated that the modest temperature elevation during labour is a risk factor for caesarean and assisted vaginal delivery [20]. Over 93% of women in both groups didn´t receive any measurement of temperature during labour management in this study.

Vaginal examination (VE) is one of the core procedures during childbirth to obtain necessary information about cervical dilatation, effacement, foetal head position and status of membranes to make timely decisions on relevant obstetric interventions. While this study revealed 1.4 times higher risk of having intrapatum stillbirth among women who didn´t receive timely VE, evidence is not conclusive regarding frequency and effectiveness of this intervention during labour management [21].

The goal of induction of labour is to achieve a vaginal delivery when the benefits of expeditious delivery outweigh the potential risk of continuing pregnancy [22]. This study revealed that 14% of women in the intrapartum stillbirth group against 15% in the livebirth received induction during the index labour. This was lower than the anticipated 20% rate of induction in the developing countries context [23]. However, the difference between the two groups was not statistically significant.

Episiotomy is a surgical cut of the perineum to increase the diameter of the pelvic outlet which might be undertaken to expedite vaginal delivery in case foetal compromise or prolonged labour were diagnosed [24]. More women in the intrapartum stillbirth group (72.4%) did not receive episiotomy care compared to women in the livebirth group (67.6%) where the difference was statistically significant (p<0.05). A study conducted in the US showed relatively fewer incidence of episiotomy among women in the stillbirth group (2%). However, this result was lower compared to their expected 25% national estimate [25]. Evidence on the importance of episiotomy in reducing the adverse pregnancy outcomes including stillbirth is inconclusive [26]. However, the practice is commonly cited in the obstetric textbooks and being exercised by many skilled birth attendants. Findings from this study showed 1.5 times higher risk of experiencing intrapartum stillbirth among women who didn´t receive episiotomy.

This study revealed that only 7.3% of women in the intrapartum stillbirth group and 8% in the livebirth group received care related to instrumental delivery. However, this finding was much higher than a study from Nepal where assisted delivery care was provided to only 2.2% in the stillbirth and 2.4% in the livebirth categories [17]. Moreover, data from the current study did not establish any benefit of instrumental delivery against intrapartum stillbirth as the difference was not statistically significant.

## Conclusion

Findings on intrapartum obstetric interventions including monitoring of foetal heartbeat, maternal vital signs, uterine contraction and cervical dilatation during labour showed that the quality and intervals of obstetric care interventions provided to women in the intrapartum stillbirth group were inferior compared to livebirth groups. For instance, proportionally more women in the livebirth groups than intrapartum stillbirth received intrapartum care related to monitoring of uterine contractions and blood pressure in timely manner, the differences being statistically significant. Similarly, women in the intrapartum stillbirth group received substandard care regarding timely assessment of foetal decent, cervical dilatation, labour induction and episiotomy care compared to women in the livebirth group. Furthermore, obstetrical complications including obstructed labour, eclampsia and preeclampsia were more common among women in the intrapartum stillbirth group. All these results suggest that poor quality of obstetric care during labour and childbirth can be a risk factor for intrapartum stillbirth. The medical records reviewed under this study didn´t contain the exact timing of intrapartum stillbirth thereby limiting the possibility to determine the appropriateness of the obstetric interventions and to draw any conclusion regarding cause-effect relationship between the variables. More rigorous and prospective studies are recommended to fill this evidence gap.

### What is known about this topic

- The magnitude of intrapartum stillbirth globally and in different regions of the world;
- High proportion of intrapartum stillbirth can be prevented through quality and timely intrapartum care.

### What this study adds

- Women who had inconsistent monitoring of uterine contractions during labour were 1.5 times more likely to experience intrapartum stillbirth in the study setting;
- While the benefit of episiotomy as an obstetric care intervention seems inconclusive in many literatures, this study reported a 1.5 times higher risk of experiencing intrapartum stillbirth among women who didn´t receive episiotomy.
